# Major Scientific Hurdles in HIV Vaccine Development: Historical Perspective and Future Directions

**DOI:** 10.3389/fimmu.2020.590780

**Published:** 2020-10-28

**Authors:** Tiza Ng’uni, Caroline Chasara, Zaza M. Ndhlovu

**Affiliations:** ^1^KwaZulu-Natal Research Institute for Tuberculosis and HIV (K-RITH), Nelson R. Mandela School of Medicine, University of KwaZulu-Natal, Durban, South Africa; ^2^Ragon Institute of Massachusetts General Hospital, Massachusetts Institute of Technology, and Harvard University, Cambridge, MA, United States

**Keywords:** HIV, history of HIV-1 vaccines, efficacy trials, HIV prevention, HIV-1 vaccine design

## Abstract

Following the discovery of HIV as a causative agent of AIDS, the expectation was to rapidly develop a vaccine; but thirty years later, we still do not have a licensed vaccine. Progress has been hindered by the extensive genetic variability of HIV and our limited understanding of immune responses required to protect against HIV acquisition. Nonetheless, valuable knowledge accrued from numerous basic and translational science research studies and vaccine trials has provided insight into the structural biology of the virus, immunogen design and novel vaccine delivery systems that will likely constitute an effective vaccine. Furthermore, stakeholders now appreciate the daunting scientific challenges of developing an effective HIV vaccine, hence the increased advocacy for collaborative efforts among academic research scientists, governments, pharmaceutical industry, philanthropy, and regulatory entities. In this review, we highlight the history of HIV vaccine development efforts, highlighting major challenges and future directions.

## Introduction

The HIV/AIDS epidemic remains a major global health challenge and continues to exert significant strain on healthcare resources in sub-Saharan Africa. According to the UNAIDS, globally, approximately 37.9 million people were living with HIV infection in 2018. In addition, there were 1.7 million new infections with approximately 770,000 AIDS-related deaths in the same year despite widespread rollout of antiretroviral therapy (ART) (1, 2). The global HIV incidence-to-prevalence ratio of 0.05 indicates that the number of HIV-infected people will continue to rise unless more effective preventive strategies are employed to reduce transmission (3). There is broad scientific consensus that the most effective approach to control and eventually end the HIV epidemic is to develop a preventive AIDS vaccine that is safe, effective, cost efficient, and easily accessible worldwide (4). Regrettably, despite over 30 years of rigorous HIV research and numerous vaccine trials, there is no licensed HIV vaccine currently on the market (5). The aim of this review is to discuss past and present approaches to vaccine development and clinical trials to date. The review also highlights current gaps in knowledge and proposes new directions and novel strategies toward developing an efficacious preventive HIV vaccine.

## Challenges in HIV Vaccine Development

The development of potent antiretroviral therapies, now delivered as a single pill once a day, has transformed HIV infection into a clinically manageable chronic disease. Globally, over 19 million people are now on life-long treatment, and test-and-treat strategies and oral pre-exposure prophylaxis (PrEP) could further reduce HIV transmissions. However, despite these remarkable advances, prolonged combined antiretroviral therapy (cART) mediated suppression of plasma viral loads to undetectable levels does not eradicate the virus, which often rapidly rebounds upon treatment interruption. In addition, while cART has decreased mortality and morbidity among people living with HIV (PLWH), long-term cART treatment is associated with increased occurrence of a range of serious non-AIDS events (SNAEs). These SNAEs include cardiovascular diseases, cancer, liver disease, long-term peripheral and central nervous system complications, renal and metabolic disorders, and osteoporosis (6). The many logistical limitations and cost challenges that come with providing life-long care to those living with HIV highlight the need for a preventive HIV vaccine (7). Desirable attributes of an HIV vaccine include elicitation of long-lasting all-round protection with a limited number of doses administered to the patient, the vaccine should also be affordable, easy to administer, and easy to store without the need of a cold chain. The HIV vaccine can either be preventive or therapeutic, which means it can either block HIV infection or could be used to treat HIV infected individuals (8).

Over the years, the greatest challenge in developing an effective HIV vaccine has been the high rate of mutation and recombination during viral replication (9). The enormous genetic diversity of HIV is mainly driven by the high rate of variability of the viral envelope (Env) glycoprotein, which ironically happens to be the main target of neutralizing antibodies (10). The HIV genome contains nine genes which encode 16 proteins including the major structural proteins Gag, Pol, and Env; accessory proteins Nef, Vif, Vpu, and Vpr; and regulatory proteins Tat and Rev. HIV diversity, which is mainly generated by the error prone viral reverse transcriptase, has various implications for disease progression and responses to ART (11). The high mutation rates of approximately 1–10 mutations per genome per replication cycle, extensive conformational adaptability, and massive glycan shielding of the Env enable the virus to evade the effects of neutralizing antibodies and other immune responses (12). Nonetheless, despite the high rate of variability, it has progressively been shown that polyvalent HIV vaccines can be developed and used to target conserved domains on the viral envelope (13). Most current efforts are aimed at inducing broadly neutralizing antibodies (bNAbs), which can neutralize the majority of HIV strains. The ability of bNAbs to neutralize a wide spectrum of HIV strains (broad cross-reactivity) is a major advantage (14). Moreover, the safety and remarkable antiviral activity of highly potent HIV specific bNAbs have been demonstrated in pre-clinical and clinical trials (15).

Beside high viral mutation and recombination rates, extraordinary worldwide genetic diversity is yet another hurdle to the development of a vaccine. HIV is composed of 4 groups: M (main), O (outlier), N (non-M/non-O), and P (pending). Group M is further subdivided into 9 subtypes/clades denoted by the letters A, B, C, D, F, G, H, J, and K. It has been shown that amino acid variations within subtypes can be as high as 30% with those between subtypes reaching as high as 42%. These amino acid variations are based on the subtypes and region of the genome being examined (16, 17). The difficulty of developing a universal vaccine is further compounded by the fact that 10–20% of HIV infected people in several parts of Africa, are infected with two or more viral variants (subtypes and recombinant forms) that circulate in these regions (18).

Other challenges that impact HIV vaccine development include an incomplete understanding of the correlates of immune protection, lack of appropriate animal models, and limited investments by the pharmaceutical industry (19, 20). In addition, most traditional immunogen delivery systems are unable to induce potent and long-lasting immunity against HIV and the traditional live attenuated or whole-inactivated virus methods, employed in the design of measles, mumps, and rubella vaccines, are not appropriate for HIV due to legitimate safety and regulatory concerns associated with the risk of permanent integration of proviral HIV DNA into the host genome (4, 21).

## The Development of Early HIV Vaccines

Following the isolation of HIV as a causative agent for AIDS in 1983–1984, numerous vaccine prototypes have to date failed to protect against HIV infection (22, 23). In 1984, it was speculated that an HIV vaccine would be developed and available for testing in approximately two years (24). In hindsight, it is clear that researchers underestimated the complexity of such a scientific undertaking. We now know that HIV is unlike any other viral disease for which effective vaccines were empirically developed (25, 26). Given the risk of irreversible HIV integration into the human genome, rational design of subunit vaccines is the only viable option. The introduction of recombinant DNA technologies in the mid-1980s presented the best approach to develop a safe and effective HIV vaccine. This idea was derived from the hepatitis B model for which the hepatitis B surface antigen was successfully cloned and expressed in yeast cells, thus allowing for a new recombinant hepatitis B vaccine to be manufactured and licensed in 1986 (24). Using the hepatitis B recombinant DNA model, HIV researchers developed a subunit vaccine based on genetically engineered antigens representing the outer envelope glycoproteins of HIV. The design was based on previous vaccine approaches that used virus subunits, synthetic peptides, and vaccinia-vector vaccines in animal models (27–29). Rapid developments in the molecular biology of HIV, such as the identification of the major viral structural proteins (30) and the cloning and sequencing of the HIV genome (31), benefited initial vaccine development efforts. However, the genetic variability of HIV still remained the biggest obstacle to the success of these vaccine development efforts (32).

## HIV Vaccines Based on the Induction of Neutralizing Antibodies

Initially, scientists believed that neutralizing antibodies would be adequate to protect against HIV infection (33) and many of the HIV vaccines in this category were designed to primarily target the envelope glycoproteins, gp120 or gp160 (24). The first experimental immunization of humans against HIV/AIDS was done in Zaire (now the Democratic Republic of Congo) by Zagury and colleagues in 1986 (34). A small group of Zairians were vaccinated with a vaccinia vector (a recombinant HIV-vaccinia virus) expressing gp160, an envelope glycoprotein. The purpose of the study was to assess whether vaccination could induce neutralizing antibodies and cytotoxic T lymphocyte (CTL) responses in HIV negative individuals. Administration of the recombinant vaccinia virus vaccine by scarification elicited weak humoral and cellular immune responses that were boosted by four additional immunizations to achieve anamnestic humoral responses and cellular responses, which persisted for more than a year (35). Following this pioneering in human experimental HIV/AIDS vaccine trial, over 250 clinical trials have been carried out, with the majority being early-phase trials (phase 1 or 2) (36). Approximately 140 of these trials were conducted in the United States with several others being carried out in African countries and Thailand (24). Major vaccine trials have been completed and timelines are shown in **Table 1**.

**Table 1:** Illustration of completed and documented HIV vaccine trials.

Vaccine Trial	Year	Site	Target group	Vaccine	Immune response	Result	Reference
VaxSyn	1987	Canada (Clade B)	72 adults	Recombinant envelope glycoprotein subunit (rgp160) of HIV	Neutralizing antibodies were detected	No vaccine efficacy	(37)
HIVAC-1e	1988	USA (Clade B)	35 male adults	Recombinant vaccinia virus designed to express HIV gp160	Vaccine was unable to confer protection against HIV	No vaccine efficacy	(38)
Vax004	1998–2002	North America (Clade B)	5,417 MSM and 300 women	AIDSVAX B/B gp120 with alum	Vaccine was unable to confer protection against HIV	No vaccine efficacy	(39, 40)
Vax003	1999–2003	Thailand (Clade B/E)	2,545 men and women IDUs	AIDSVAX B/E gp120 with alum	Vaccine was unable to confer protection against HIV	No vaccine efficacy	(39, 40)
HVTN 505	2009–2013	United States (Clade B)	2,504 men or transgender women who have sex with men	Three vaccinations with DNA encoding HIV clade B *gag*, *pol* and *nef* as well as *env* from HIV clades A, B and C followed by an Ad5 vector-based vaccine encoding clade B *gag* and *pol* as well as *env* from clades A, B and C	Vaccine was unable to prevent infection or decrease viral load in vaccinated volunteers	No vaccine efficacy	(41, 42)
STEP/HVTN 502 trial	2004–2007	North America the Caribbean South America, and Australia (Clade B),,	3,000 MSM and heterosexual men and women	MRKAd5 HIV-1 *gag*/*pol*/*nef* trivalent vaccine	Vaccine was unable to confer protection against HIV	No vaccine efficacy	(43, 44)
Phambili/HVTN 503 trial	2003–2007	South Africa (Clade C)	801 adults	rAd5 (*gag*/*pol*/*nef*)	Vaccine was unable to confer protection against HIV	No vaccine efficacy	(24)
RV144	2003–2009	Thailand (Clade B)	16,402 community-risk men and women	ALVAC-HIV (vCP1521) and AIDSVAX B/E vaccines	IgG antibody avidity for Env in vaccine recipients with low IgA	31.2% vaccine efficacy at 42 months	(45, 46)
HVTN 305	2012–2017	Thailand (Clade B/E)	162 women and men	ALVAC-HIV and AIDSVAX B/E		No vaccine efficacy	(47)
HVTN 306	2013–2020	Thailand (Clade B/E)	360 men and women aged 20–40 years	ALVAC-HIV and AIDSVAX B/E	Vaccine was unable to confer protection against HIV	No vaccine efficacy	(48)
HVTN 097	2012–2013	South Africa (Clade B/E)	100 black Africans (men and women) aged 18–40 years	ALVAC-HIV (vCP1521) and AIDSVAX B/E	Induction of CD4^+^ T cells directed to HIV-1 Env	No vaccine efficacy	(49)
HVTN 100	2015–2018	South Africa (Clade C)	252 men and women	ALVAC-HIV (vCP2438) and bivalent subtype C gp120/MF59	CD4^+^ T-cell responses and gp120 binding antibody responses	No vaccine efficacy	(50)
HVTN 702	2016–2020	South Africa (702Clade C)	5,400 men and women	ALVAC-HIV (vCP2438) and bivalent subtype C gp120/MF59	Vaccine was unable to confer protection against HIV	No vaccine efficacy	(51)

MSM, men who have sex with men; IDUs, IV drug users.

### VaxSyn and HIVAC-1e Vaccines

The first HIV vaccine trial carried out in the US investigated a recombinant envelope glycoprotein (rgpl60), VaxSyn, created in a baculovirus-insect cell system. This trial evaluated the safety and immunogenicity of a rgpl60 candidate vaccine in 72 healthy, HIV-negative adults. The vaccine recipients were randomly assigned to one of four groups to receive intramuscular injections of 40 or 80 µg of rgpl60, 10 µg of hepatitis B vaccine, or placebo in three doses (on days 0, 30, and 180) with an optional fourth dose on day 540. The placebo and hepatitis B vaccine groups served as control groups (37). Results showed that the vaccine was safe and well tolerated. It was observed that vaccines recipients receiving 40 or 80 µg of rgpl60 displayed mostly weak serum antibody responses to HIV envelope proteins. However, antibody titers noticeably increased after the third dose and declined over an 18-month period. The administration of a fourth dose resulted in homologous neutralizing activity and enhanced complement-mediated antibody-dependent activity in some vaccine recipients (37). Other studies showed that the administration of VaxSyn at a dose of 640 µg resulted in increased immunogenicity and higher rates of homologous neutralizing antibody responses, despite the titer being low (52) but failed to elicit sufficient protective neutralizing antibodies (37). After the VaxSyn trial, numerous envelope proteins were evaluated in 35 phase I trials, between 1988 and 2003. Collectively, these vaccine constructs induced binding and neutralizing antibodies that were remarkably durable and also primed CD4^+^ T cell responses but no apparent CTL responses (24).

In 1988, a second recombinant vaccinia virus designed to express HIV gp160 (HIVAC-1e) entered phase 1 clinical trials in the US. The study enrolled 35 HIV-negative males (31 of whom had prior smallpox immunisations and 4 of whom were vaccinia naive). Study participants were randomly allocated to receive either standard New York strain vaccinia virus or HIVAC-le. Results showed that vaccinia-naive subjects shed virus from the vaccination site for longer and at a higher titer than the vaccinia-primed individuals. It was also observed that *in-vitro* T-cell proliferative responses to one or more HIV antigen preparations developed in some vaccinia-primed subjects inoculated with HIVAC-1e. However, T-cell responses were short-lived, and no HIV-specific antibodies were detectable. Nonetheless, two vaccinia-naive subjects vaccinated with HIVAC-1e showed robust T-cell responses to homologous and heterologous whole virus stains and to the recombinant gp160 protein, which were detectable for over a year. HIV Env specific antibodies also developed in both subjects. Interestingly, despite HIVAC-1e inducing transient and robust T-cell responses, it failed to produce antibodies against HIV infection in other subjects (24, 38). This led researchers to posit that antibody responses could be improved by priming with a recombinant vaccinia vector expressing the HIV-1 envelope and later boosting with an envelope protein.

In 1991, a combined approach was used in a phase 1 vaccine trial conducted in the US. This trial primed with HIVAC-1e and later boosted with VaxSyn. The prime-boost approach, used in several clinical trials, significantly enhanced both humoral and cellular immune responses and induced neutralizing antibodies (24, 53, 54). Despite these promising results, the use of vaccinia virus vectors raised several concerns. For instance, there was notable decrease in the immunogenicity of the vector in individuals previously vaccinated against smallpox (55, 56). In addition, there were concerns that administering the replicating vaccinia to immunosuppressed individuals could result in severe disease (55, 56). These concerns led to the development of non-replicating poxvirus vectors in the early 1990s, based on two models, namely, a highly attenuated vaccinia virus (NYVAC) or an avian poxvirus, Canarypox (ALVAC), that is not able to replicate in mammalian cells (57, 58).

### ALVAC-HIV Vector Vaccine

In 1993, an ALVAC-vector HIV vaccine, vCP125, expressing gp160 was tested alone or as a prime-boost combination with an adjuvanted gp160 subunit. The results revealed that the ALVAC-HIV vaccine significantly primed the neutralizing antibody response of the protein boost and induced CTL activity (59). Other ALVAC-vectors (vCP205, vCP300, vCP1433, vCP1452, and vCP1521) were developed not only to express the HIV envelope but also to express *gag* and other HIV genes to induce broader cell-mediated immune responses (60, 61). In 1999, the second ALVAC-1 vector HIV vaccine was tested in Ugandan infants born to HIV infected mothers in a randomized placebo-controlled double-blind phase 1 trial. The vaccine construct expressed multiple genes such as *gp120*, the anchor region of *gp41*, *gag*, and *protease* (62–64). Even though the vaccine design was based on clade B genes, the researchers justified testing it in Uganda where clades A and D dominate based on the widespread cross-clade cellular immune responses observed in pre-clinical studies (63, 64). The results showed that the vaccine was safe in infants. Notably, vCP1521 was the prime employed in the Thai RV144 trial (discussed below) (45, 61).

### VAX003 and VAX004 Efficacy Trials

1994 saw the emergence of two possible vaccine candidates (consisting of formulations of bivalent recombinant gp120 and alum) that were used in efficacy trials. These vaccine concepts were advanced to efficacy trials because they conferred protection to chimpanzees following HIV challenge and were safe and immunogenic in phase 1/2 clinical trials in humans (65, 66). The first two efficacy trials were carried out, from 1998 to 2003, by VaxGen in North America (VAX004; ClinicalTrials.gov Identifier: NCT00002441) and Thailand (VAX003; ClinicalTrials.gov Identifier: NCT00006327) (67–69). Based on the knowledge gained regarding genetic variability of HIV strains and the ability to use various co-receptors, the two initial candidate HIV vaccines were redesigned as bivalent gp120 vaccines (AIDSVAX B/B) for the North American trial and (AIDSVAX B/E) for the Thailand trial (the AIDSVAX B/E gp120 boost was also used in the RV144 trial) (45, 70). The two redesigned gp120 vaccines were derived from R5 and X4 strains (HIV strains using CCR5 and CXCR4 co-receptors, respectively) (71). The VAX004 efficacy trial recruited 5417 volunteers who were mainly men who have sex with men (MSM) in North America, and the VAX003 trial recruited 2545 volunteers comprising intravenous injection drug users in Bangkok, Thailand. Unfortunately, in 2003, data analysis revealed that the two vaccines did not prevent HIV acquisition and did not ameliorate disease (39, 40).

## Vaccines Designed to Stimulate T Cell Immunity to HIV

Multiple failures in antibody-based vaccines such as the VaxGen gp120 trials prompted the HIV vaccine field to begin pursuing T cell-based vaccines. There was a growing body of evidence demonstrating that CD8^+^ T cell responses play a key role in controlling HIV infection. Animal studies showed that depletion of CD8^+^ T cells in acute infection led to loss of virus control (72–75). Human studies also showed that the emergence of HIV-specific CD8^+^ T cell responses coincided with the decline of viral load to a set point (76). Discovery of CD8^+^ T cell driven viral mutations was another piece of evidence highlighting the importance of CD8^+^ T cell responses in immune mediated control of HIV infection. Moreover, cumulative data showing CD8^+^ T cell responses are largely responsible for spontaneous control of viremia for prolonged periods in the absence of medication, provided the impetus for the vaccine field to seriously pursue the development of T cell-based HIV vaccines (72, 77, 78). The candidate model vaccine vectors used for T cell-based vaccines were live recombinant viral vectors, mainly pox and adenovirus vectors (particularly the replication-defective adenovirus 5 [Ad5]), as well as DNA vaccines (79–81).

### T Cell-Based Phase 2b Efficacy Trials

The first large T cell-based vaccine trial, the STEP trial also called HVTN 502 trial (ClinicalTrials.gov Identifier: NCT00095576), was a phase 2b multicentre, double-blind, randomized, placebo-controlled “test-of-concept” study that tested the efficacy of the MRKAd5 HIV-1*gag*/*pol*/*nef* vaccine. In December 2004, 3,000 participants were enrolled from Australia, Brazil, Canada, the Dominican Republic, Haiti, Jamaica, Peru, Puerto Rico, and the US where HIV subtype B is prevalent. The aim of the STEP trial was to determine whether the vaccine could prevent HIV infection, decrease the viral load in HIV-infected individuals, or both. Participants were randomly assigned to either vaccine or placebo groups, in a 1:1 ratio, to receive 3 injections of MRKAd5 HIV-1 *gag*/*pol*/*nef* vaccine or placebo (43, 44). The MRKAd5 HIV-1*gag*/*pol*/*nef* vaccine was also tested in a phase 2b trial (the Phambili/HVTN 503 trial; ClinicalTrials.gov Identifier: NCT00413725) in 801 adult South Africans. The goal of this trial was to evaluate the effectiveness of the vaccine in preventing infection in Southern Africa, where HIV subtype C is predominant. Unfortunately, vaccination and enrolment into both the STEP and Phambili trials were terminated in September 2007 following preliminary assessment that demonstrated no efficacy (24, 82). Furthermore, multivariate analysis of baseline risk factors revealed that the vaccination resulted in increased risk of HIV infection in some volunteers. In 2008, the final results of the trial were published, showing that cell-mediated immune responses elicited by this vaccine did not prevent HIV infection or blunt peak viral load (44). Additionally, there were slightly more HIV infections in the vaccine group compared to the placebo group in uncircumcised men with Ad5-neutralizing antibodies (43).

HVTN 505 (ClinicalTrials.gov Identifier: NCT00865566) was the next T cell-based phase 2b randomized, placebo-controlled, efficacy trial initiated in 2009 to test a potential DNA-primed vaccine in 2504 men or transgender women who have sex with men using a prime-boost regimen. The regimen consisted of three vaccinations with DNA encoding HIV clade B *gag*, *pol*, and *nef* as well as *env* from HIV clades A, B and C followed by an Ad5 vector-based vaccine encoding clade B *gag* and *pol* and *env* from clades A, B, and C (41, 42, 83). The experimental group received a DNA-primed vaccine injection on days 0, 28, and 56 followed by an Ad5 vector-based vaccine injection on day 168. The placebo group received placebo injections on days 0, 28, 56, and 168. Unfortunately, the trial was prematurely terminated after 47 months because interim analysis showed that the vaccine was not able to prevent infection or decrease viral load in vaccinated volunteers. Moreover, there was a slight increase in breakthrough infections in vaccine recipients compared to placebo controls (24).

The failure of conventional approaches to generate CD8*^+^* T cell-based vaccines prompted the scientific community to search for new approaches to HIV vaccine design. In 2008, NIAID encouraged researchers to go back to basics. A meeting convened by NIAID endorsed the expansion of research agenda to answer both basic science and novel vaccine design questions (24).

## A Vaccine Against HIV-1 Is Possible

The multiple setbacks in the HIV vaccine field led to substantial discussions regarding the optimal path toward a vaccine. The unexpected success of the RV144 trial (ClinicalTrials.gov Identifier: NCT00223080) showing modest but significant vaccine-induced protection (31.2% by 42 months) against HIV acquisition provided renewed hope that an HIV vaccine is possible. The RV144 was a randomized, double-blind phase 3 efficacy trial that utilized a recombinant canarypox vector vaccine, ALVAC-HIV (vCP1521), expressing Env (clade E), group-specific antigen (Gag) (clade B), and protease (Pro) (clade B), and an alum-adjuvanted AIDSVAX B/E and a bivalent HIV glycoprotein 120 (gp120) subunit vaccine (45, 46, 84). Vaccine recipients were given four priming injections of ALVAC-HIV (vCP1521) at months 0, 1, 3, and 6 with two booster injections of AIDSVAX B/E administered at months 3 and 6 (70). Immune correlates analyses revealed that the regimen induced HIV-specific humoral and cellular immune responses which resulted in reduced risk of HIV infection (inversely correlated with risk of HIV infection). Vaccine-induced responses included IgG antibodies binding to the HIV Env variable loops 1 and 2 (V1V2) and antibody-dependent cellular cytotoxicity (ADCC) in vaccine recipients with low IgA (70, 85–87). The magnitude and polyfunctionality of Env-specific CD4^+^ T cells were also later shown to play a role in reducing the risk of HIV infection (70, 85–87).

### Follow-Up Studies Based on the RV144 Trial

To date, only the RV144 trial has shown modest efficacy of 31.2% 42 months after the final vaccination. Rapid decline in protective antibody levels in most vaccine recipients led to the proposition that late boosts would induce durable protective immune response (88). Therefore, late boost studies (RV305 and RV306) were designed to assess immune responses generated in newly boosted vaccine recipients compared to RV144 vaccine recipients (47, 48).

#### RV305 Phase 2 Trial

The RV305 trial (ClinicalTrials.gov Identifier: NCT01435135) was a randomized, placebo-controlled, double-blind study conducted to investigate whether late boosts with ALVAC-HIV (vCP1521) or AIDSVAX B/E, administered either alone or in combination could enhance immune correlates of protection. The trial re-enrolled 162 healthy, HIV-negative Thai RV144 vaccine recipients to receive 2 additional boosts given 6–8 years after RV144 vaccination. Study participants were randomized into one of three groups to receive either vaccine or placebo. Group 1 received ALVAC-HIV and AIDSVAX B/E, group 2 received AIDSVAX B/E, and group 3 received ALVAC-HIV, or placebo, at weeks 0 and 24 (47). Results showed that plasma immunoglobulin G (IgG), IgA, and neutralizing antibody responses at week 2 were all significantly higher in groups 1 and 2 compared to the responses observed 2 weeks following the last RV144 vaccination. The boost also resulted in higher antibody titers against various Env antigens (such as gp120 and V1V2) which were above the levels observed at the peak RV144 vaccine time point in plasma and mucosal secretions (47, 89). While the antibody titers increased following the first boost, they did not increase following the second boost in RV305 vaccine recipients. Moreover, the administration of late boosts did not result in lasting antibody responses as they rapidly declined after boosting in all groups (47). Overall, it was concluded that administering late boosts to RV144 vaccine recipients (6–8 years following their last vaccination) was safe and well tolerated. In addition, it was observed that despite AIDSVAX B/E alone or in combination with ALVAC-HIV generating higher humoral and CD4^+^ T cells responses in RV305 vaccine recipients, these responses were short-lived and subsequent boosts did not increase their magnitude (47).

#### RV306 Phase 2 Trial

The RV306 trial (ClinicalTrials.gov Identifier: NCT01435135) was a randomized, placebo-controlled, double-blind study conducted at three clinical sites in Thailand. This study was designed to determine whether boosting the RV144 regimen at either month 12, 15, or 18 following initial vaccination would improve the quality, magnitude or duration of humoral, cellular, and mucosal responses. In addition, it was designed to also establish the optimal boosting interval for further clinical development (48). The study enrolled 367 healthy, HIV-negative individuals and randomly allocated them to one of five groups to receive vaccine or placebo. All groups received the original RV144 vaccination regimen at months 0, 1, 3, and 6 as follows: ALVAC-HIV at months 0 and 1 followed by either ALVAC-HIV and AIDSVAX B/E or placebo at months 3 and 6. Group 1 received only the RV144 series and no additional boost, group 2 received additional ALVAC-HIV and AIDSVAX B/E or placebo at month 12, group 3 received AIDSVAX B/E alone or placebo at month 12, group 4a received ALVAC-HIV and AIDSVAX B/E or placebo at month 15, and group 4b received ALVAC-HIV and AIDSVAX B/E or placebo at month 18. No serious vaccine-related adverse events were reported across active groups. Furthermore, it was observed that groups with late boosts (groups 2, 3, 4a, and 4b) had increased peak plasma IgG-binding antibody levels against gp70 V1V2 relative to group 1 vaccine recipients with no late boost. It was also observed that boosting at month 12 (groups 2 and 3) did not increase gp120 responses compared with the peak responses after the RV144 priming regimen at month 6, but boosting at month 15 (group 4a) improved responses to gp120 A244gD–D11 and boosting at month 18 (group 4b) improved responses to both gp120 A244gD–D11 and gp120 MNgD–D11. In addition, boosting at month 18 versus month 15 resulted in a significantly higher plasma IgG response to gp120 antigens but not gp70 V1V2 antigens. It was further observed that CD4 functionality and polyfunctionality scores following stimulation with HIV-1 Env peptides (92TH023) increased with delayed boosting. Additionally, the results showed that groups with late boosts had increased functionality and polyfunctionality scores relative to vaccine recipients with no late boost. Collectively, these results implied that additional boosting of the RV144 regimen with longer intervals between the initial vaccination and late boost could improve vaccine efficacy (48).

The promising results of RV144 clinical trials prompted the need to assess its efficacy against other clades (3, 90). RV144 originally designed to protect against HIV clade CRF01_AE BE infection in Thailand was modified to target HIV clade C. Therefore, HVTN designed and conducted a series of clinical trials including, HVTN 097, HVTN 100 and HVTN 702 (3).

### HVTN 097 Phase 1b Vaccine Trial

The first vaccine concept tested by HVTN based on the RV144 concept was the HVTN 097 trial (ClinicalTrials.gov Identifier: NCT02109354), which was a randomized, controlled, double-blind phase 1b study done in South Africa (49). The trial was designed to assess the safety and immunogenicity of the vaccine regimen in healthy, HIV-uninfected South African adults. The regimen consisted of two prime doses of the experimental canarypox HIV vaccine ALVAC-HIV (vCP1521) followed by two booster shots of the AIDSVAX B/E. Study participants were randomized into three groups, in a 3:1:1 ratio, to receive the vaccine combined with tetanus and hepatitis B immunizations, the vaccine only or placebo. The tetanus and hepatitis B immunizations were included to assess possible cross-correlates of immune responses to HIV vaccine, however no significant differences in HIV immune responses were observed indicating that subsequent results were solely due to immune responses to HIV (49). The prime-boost vaccine regimen induced mostly Env-specific CD4^+^ T cell responses at significantly higher levels compared to RV144 vaccine recipients (RV144 = 36.4%; HVTN 097 = 51.9%). IgG antibodies recognizing the V1V2 region and the IgG3 binding antibody responses to both gp120 and V1V2 antigens were also significantly higher among HVTN 097 vaccine recipients relative to RV144 recipients. ADCC antibody responses were also higher in HVTN 097 than in RV144, 72.6% (53 of 73) and 58.5% (114 of 195), respectively. These favourable results provided compelling rationale for conducting larger clinical trials in South Africa (49).

### HVTN 100 Phase 1/2 Preventative Vaccine Trial

The HVTN 100 (ClinicalTrials.gov Identifier: NCT02404311) phase 1/2 randomized, controlled, double-blind study, was also conducted in South Africa to evaluate the safety, tolerability, and immunogenicity of the new modified vaccine regimen for subsequent efficacy testing. It consisted of an ALVAC-HIV (vCP2438) vector, expressing HIV Env gp120 (clade C ZM96), Env gp41, Gag, and Pro (all clade B) as well as a MF59-adjuvanted bivalent subtype C gp120 protein vaccine. The MF59 adjuvant was used to boost neutralizing antibodies and T cell responses (50). Enrolment took place from February to May 2015. Vaccine recipients received ALVAC-HIV (vCP2438) vector intramuscular (IM) injections at months 0 and 1 followed by co-administrations of ALVAC-HIV (vCP2438) and MF59-adjuvanted bivalent subtype C gp120 at months 3, 6, and 12 (50). The vaccine induced greater frequency of IgG3 responses to Env gp120, significantly higher CD4^+^ T-cell responses and gp120 binding antibody responses compared to the RV144 regimen. Importantly, the IgG response exceeded the expected 63% threshold required for 50% vaccine efficacy that was calculated using a V1V2 correlate of protection model. Therefore, the HVTN 100 vaccine regime was advanced to a phase 2b/3 efficacy trial (HVTN 702) (50). It is important to note that despite the frequency of IgG3 and V1V2 antibody responses in HVTN 100 exceeding levels that were modeled to be required for protection, the correlate of reduced risk in RV144 was not response rate but rather the level of the antibodies (HIV-specific antibody responses resulting in reduced risk of HIV acquisition) (70, 85–87). Therefore, this criterion used for advancement to the phase 2b/3 HVTN 702 trial was not consistent with the findings of the RV144 trial.

### HVTN 702 Phase 2b/3 Efficacy Trial

The HVTN 702 trial (ClinicalTrials.gov Identifier: NCT02968849) was a randomized, controlled, double-blind study conducted at 14 sites in South Africa from 2016 to 2020. The main objective was to assess the efficacy, safety, and tolerability of the ALVAC-HIV (vCP2438) plus bivalent Subtype C gp120/MF59 prime-boost vaccine regimen. The study enrolled 5407 HIV-uninfected sexually active individuals (both men and women), aged 18–35 years, and randomly allocated them to the vaccine or placebo arm. Vaccine recipients received an IM injection of ALVAC-HIV (vCP2438) at months 0 and 1 followed by co-administrations of ALVAC-HIV (vCP2438) + Bivalent Subtype C gp120/MF59 at months 3, 6, and 12 (51). The vaccine regimen employed in the HVTN 702 was modified to improve the efficacy and durability of the immune responses compared to RV144. Firstly, ALVAC-HIV vaccine construct contained clade C HIV genetic inserts to match those predominantly found in South Africa whereas the RV144 ALVAC contained clade B and E genetic inserts (those predominantly found in Thailand). Secondly, the boost in the HVTN 702 trial was a clade C genetically engineered HIV gp120 protein that was co-formulated with the MF59 adjuvant, whereas RV144 utilized the adjuvant alum. Thirdly, vaccine candidates in the HVTN 702 trial received five injections administered over a 12-month period (0, 1, 3, 6, and 12), whereas the RV144 vaccine candidates received four injections over a 6-month period (0, 1, 3, and 6). It was believed that a fifth dose at month 12 would potentially result in an extended protective effect (45, 51). Unfortunately, the trial was stopped on 23 January 2020 following an interim analysis by an independent data and safety monitoring board (DSMB). The DSMB analysed data from 2694 vaccine recipients and 2689 placebo recipients and discovered that 129 HIV infections occurred among the vaccine recipients, and 123 HIV infections occurred among the placebo recipients (91, 92). These findings indicated that the vaccine was not effective in preventing HIV infection and the DSMB recommended discontinuation of further vaccinations but allowed follow-up to continue. This was a very disappointing and unexpected result given that prior studies showed the vaccine exhibited greater immunogenicity compared to RV144. The borderline statistical significance of the RV144 results of 31.2% protective effect has led others to question the veracity of the analyses (93). Clearly, more work is needed to understand the discrepancy between immunogenicity in the HVTN 100 study and the lack of efficacy in the HVTN 702 study; this will inevitably include revisiting the RV144 analyses.

## Current Status of the HIV Vaccine Field

During the past 30 years, only a few HIV vaccine regimens have been tested in phase 2b clinical trials (3, 24, 26, 36, 94). More recently, there has been strong advocacy for adaptive clinical trials aimed at accelerating vaccine development by rapid evaluation of vaccine candidates in small human studies and rapidly advancing promising candidates to efficacy trials (95–97). The new accelerated approach has resulted in more than 100 HIV vaccine concepts being clinically tested. Similar approaches have been adopted in the accelerated development of novel coronavirus disease 2019 (COVID-19), vaccines. Collaborative efforts such as the Pox-Protein Public Private Partnership (P5) which includes private industry, government agencies, the Bill and Melinda Gates foundation, and HVTN have contributed to accelerating vaccine trials through training and establishment of vaccine testing sites across the world. Additionally, partnerships between Janssen Pharmaceutical company, academic labs, and HVTN have championed the development and testing of mosaic-based vaccines. These private-public partnerships have led to the current rich pipeline of new vaccine concepts in preclinical trials and various stages of clinical trials. Although the vaccine field is trying to accelerate the extensive pipeline of vaccine concepts to efficacy trials, the decline of HIV incidence worldwide and the wider deployment of other HIV prevention tools such as Pre- exposure prophylaxis (PrEP) has complicated vaccine testing landscape by necessitating very large, more complex, and very expensive vaccine trial designs.

### Passive Immunization Studies

For years, passive immunisation with protective antibodies has been used in the prevention and treatment of several bacterial and viral infections, subsequently influencing the current HIV vaccine field (98). The diverse mechanism of action of antibodies (through their interaction with the innate and adaptive arms of the immune system), coupled with their ability to bind and neutralize viruses, continues to make the antibody-based approach appealing to researchers (99, 100). The identification of various bNAbs with increased breadth and potency such as PG9, PG16, PGT121, PGT145, VRC01, VRC07, and 3BNC117 has provided an opportunity for their potential application in HIV vaccine research (101). Moreover, animal studies have demonstrated the protective and therapeutic properties of numerous bNAbs (102). Additionally, bNAbs have been shown to reduce viremia and delay viral rebound following ART interruption in HIV-infected individuals (103–105). However, it is unknown whether bNAbs are able to prevent HIV infection in humans (106). Hence, the need for the continued evaluation of the protective efficacy of passive immunization with bNAbs.

#### Early Passive Immunization Studies

The wealth of evidence from early passive immunization studies in animal models has revealed that the passive infusion of bNAbs resulted in protection from HIV infection (101). While several animal models have been used in passive immunisation studies, the most commonly used are Non-Human Primate (NHPs) and humanized mouse models (101). NHPs are typically infected with either simian immunodeficiency virus (SIV) or chimeric Simian/Human Immunodeficiency Virus (SHIVs), expressing the HIV Env in a SIV backbone (101). However, it has been shown that antibodies specific for the HIV Env protein are unable to neutralize SIV due to the difference in the HIV and SIV Env protein composition (102, 107). It is for this reason that SHIVs have been frequently used to infect NHPs, while humanized mouse models are directly infected with HIV in antibody protection studies (101, 102).

One of the earliest antibody protection studies in a SHIV challenge model, used polyclonal HIV IgG derived from HIV-infected chimpanzees. It was shown that the passive transfer of HIV IgG to pig-tailed macaques protected them from SHIV (based on the HIV DH12 strain) challenge (108). Another study showed that PGT121, a potent bNAb, protected monkeys from SHIV-SF162P3 challenge at serum concentrations that were lower than those previously observed (109). Several antibody protection studies evaluating the passive transfer of bNAbs in NHP models have demonstrated their ability to confer robust protection from HIV infection, even at low concentrations (97, 109–114). Furthermore, proof-of-principle studies of first-generation antibodies such as b12, that targets the CD4 binding site, in SHIV challenged monkeys have provided insight into the mechanism and durability of antibody protection (101, 115, 116). Similarly, antibody protection studies in mouse models have highlighted the protective efficacy of neutralizing antibodies (NAbs), such as b12 (117, 118) and BAT123 (119, 120). In addition, several studies have demonstrated that immunodeficient mice transplanted with human hematopoietic stem cells (hu-HSC) or bone marrow/liver/thymus (BLT) and passively immunized with bNAbs such as 2G12 (121), VRCO1 (122, 123), PG16 (124), and PG126 (125) were protected against HIV infection.

Overall, the passive infusion of SHIV challenged monkeys and HIV challenged humanized mice with bNAbs, particularly the potent second-generation antibodies, has provided evidence of their ability to effectively protect against viral infection (101). While Fc receptor binding for antibody protection has proven to be important (126, 127), the specific mechanisms by which protection is rendered are not fully understood (101, 115). Nonetheless, phase 1 human studies previously conducted to evaluate the protective efficacy of bNAbs such as 3BNC117 and VRC01 have demonstrated short-term viral control (104, 128). The use of bNAb-based vaccines in human has generated tremendous interest and clinical trials are being conducted to investigate their ability to prevent HIV infection. For instance, antibody mediated prevention (AMP) studies are being conducted to test whether VRC01 can prevent HIV infection in men who have sex with men as well as heterosexual women (106).

#### Antibody Mediated Prevention Studies

Over the years, researchers have been studying and developing bNAbs as potential HIV vaccine candidates. Subsequently, the use of bNAb-based vaccines in human trials has generated tremendous interest and clinical trials such as the Antibody Mediated Prevention (AMP) studies (HVTN 703/HPTN 081 and HVTN 704/HPTN 085) are being conducted to test whether VRC01, a potent bNAb designed to target the CD4^+^ binding site of the HIV-1 envelope glycoprotein, can prevent HIV infection in men who have sex with men as well as heterosexual women (106).

##### HVTN703/HPTN 081 Phase 2b Study

HVTN 703/HPTN 081 (ClinicalTrials.gov Identifier: NCT02568215) is a phase 2b randomized, controlled, double-blind study currently underway in sub-Saharan Africa. The study commenced in May 2016 and is expected to be completed by December 2020. This test-of-concept trial seeks to assess the safety, tolerability and efficacy of VRC01 in preventing HIV infection in healthy sexually active HIV-uninfected women. This AMP study has enrolled about 1900 HIV-uninfected sexually active women, aged 18-50 years, from several countries. Study participants, randomly allocated to one of three groups, in a 1:1:1 ratio, receive an intravenous (IV) infusion of 10 mg/kg VRC01 (low dose), 30 mg/kg VRC01 (high dose)m or placebo every 8 weeks (106, 129, 130).

##### HVTN704/HPTN 085 Phase 2b Study

The HVTN 704/HPTN 085 (ClinicalTrials.gov Identifier: NCT02716675) is another AMP study seeking to evaluate the safety, tolerability, and efficacy of VRC01 in preventing HIV-1 infection in healthy men and transgender (TG) men who have sex with men (MSM). The study commenced in March 2016 and the expected study completion date is February 2021. This study has enrolled 2701 HIV-uninfected men and transgender MSM in Brazil, Peru, Switzerland, and the United States (106, 130, 131). Participants in this study, like the HVTN 703/HPTN 081, were randomly allocated to one of three groups, in a 1:1:1 ratio, to receive a total of 10 IV infusion of 10 mg/kg VRC01 (low dose), 30 mg/kg VRC01 (high dose) or placebo every 8 weeks. The ultimate goal of the AMP trials is to identify and understand the characteristics of VRC01, such as optimal antibody concentration and effector functions, that correlate with protection against HIV infection (130).

## Current HIV Vaccine Efficacy Trials

Currently, some of the ongoing phase 2b efficacy trials include HVTN 705/HPX2008 (Imbokodo study), HVTN 706/HPX3002 and PrepVacc. Janssen Pharmaceutical in collaboration with HVTN and academic labs are testing vaccine regimens that are designed to cover different types of HIV found across the world.

### HVTN 705/HPX2008 (Imbokodo Study) Phase 2b Efficacy Trial

One such vaccine currently in efficacy trials is the HVTN 705/HPX2008 (Imbokodo study) (ClinicalTrials.gov Identifier: NCT03060629). This proof of concept study is a multicentre randomized, controlled, double-blind phase 2b/3 efficacy trial currently being conducted at 24 sites in 5 sub-Saharan African countries. This study commenced in November 2017 and is expected to be completed by May 2022. It aims to evaluate the efficacy, safety, and tolerability of a prime-boost regimen for the prevention of HIV infection. This study has enrolled 2600 healthy HIV-uninfected sexually active women, aged 18-35 years. Study participants, randomly assigned to either the experimental group or placebo group in a 1:1 ratio, received either the vaccine or placebo. The regimen consists of a tetravalent adenovirus vector vaccine, Ad26.Mos4.HIV (consisting of Ad26.Mos1.Gag-Pol, Ad26.Mos2.Gag-Pol, Ad26.Mos1.Env, and Ad26.Mos2S.Env clade C), and aluminium-phosphate adjuvanted clade C gp140 and Mosaic gp140 HIV protein vaccine. Vaccine recipients receive intramuscular (IM) injections of Ad26.Mos4.HIV at months 0 and 3 followed by IM injections of Ad26.Mos4.HIV and aluminum-phosphate adjuvanted clade C gp140 at months 6 and 12 whereas those in the placebo group will receive intramuscular injections of placebo. The primary endpoints will include; assessment of vaccine efficacy, number of participants with reactogenicity signs or symptoms, and adverse events (AEs). The secondary endpoints will include, immunogenicity, immune response biomarkers as correlates of risk of subsequent HIV acquisition, and genomic sequences of viral isolates from vaccine and placebo recipients (132, 133).

### HVTN 706/HPX3002/Mosaico Phase 3 Efficacy Trial

Another mosaic-based vaccine concept currently in clinical trials is the HVTN 706/HPX3002 (ClinicalTrials.gov Identifier: NCT03964415), or Mosaico trail. This multicentre, randomized, controlled, double-blind phase 3 efficacy trial is currently underway in Europe, North America, and South America. It commenced in October 2019 and is expected to be completed by September 2023. This study seeks to assess the safety and efficacy of the Ad26.Mos4.HIV and adjuvanted clade C gp140 and Mosaic gp140 protein vaccine prime-boost vaccine regimen in healthy, HIV-uninfected MSM and transgender people. This study has enrolled approximately 3800 participants, aged 18-60 years, and randomly allocated to receive either the vaccine or placebo as outlined in the HVTN 705/HPX2008. The primary endpoint is to assess vaccine efficacy. Secondary endpoints are, to assess the number of participants with solicited and unsolicited local and systemic adverse events (AEs), medically-attended adverse events (MAAEs) and SAEs, frequency and magnitude of HIV Env-specific humoral and cellular immune responses, antibody titers for Ad26, risky sexual behaviour, and Pre-Exposure Prophylaxis (PrEP) intake. Preliminary results reported at the International AIDS conference in Mexico city (IAS 2019), showed evidence of vaccine induced immune responses to different HIV strains circulating worldwide (133, 134).

### PrepVacc Phase 2b Trial

Finally, the PrepVacc (ClinicalTrials.gov Identifier: NCT04066881) is another multicentre, randomised, controlled, double-blind phase 2b vaccine study currently underway in Mozambique, South Africa, Tanzania, and Uganda. The study seeks to evaluate the effectiveness/efficacy of a combination of two HIV vaccine regimens (DNA/AIDSVAX and DNA/CN54gp140+ MVA/CN54gp140) with PrEP (PrEPVacc). The study commenced in January 2020 and is projected to complete in March 2023. A total of 1668 healthy HIV-uninfected adults (18-40 years) are expected to be enrolled in the study and an equal number (278) will be randomised into one of six groups (i.e. Group A, B, C, D, E, and G) to receive either the vaccine regime or placebo with PrEP. The primary endpoints will include; evaluation of HIV infection in vaccine recipients and assessment of AEs associated with receiving either vaccine or PrEP regimes which may lead to the regimes being terminated. The ultimate goal is to determine if the vaccine leads to a decrease in HIV prevalence with adequate public health significance to justify implementation of the combination vaccine regimen (133, 135). A complete list of ongoing vaccine trials is illustrated in **Table 2**.

**Table 2:** Illustration of ongoing HIV vaccine trials.

Vaccine Trial	Year	Site	Target group	Vaccine	Immune response	Result	References
HVTN 703	2016-2020	Sub-Saharan Africa (Clade C)	1900 women	VRC01 broadly neutralizing monoclonal antibody	—	Pending	(106, 130)
HVTN 704	2016-2020	Brazil Peru Switzerland, United States (Clade B),,	2701 men and transgender persons	VRC01 broadly neutralizing monoclonal antibody	—	Pending	(106, 130)
HVTN 705	2017-2022	Sub-Saharan Africa (Clade C)	2600 women	Ad26.Mos4.HIV and adjuvanted clade C gp140 and Mosaic gp140 protein vaccine	—	Pending	(132, 133)
HVTN 706	2019-2023	Europe North America and South America (Clade C),,	3800 MSM and transgender persons	Ad26.Mos4.HIV and adjuvanted clade C gp140 and Mosaic gp140 protein vaccine	—	Pending	(133, 134)
PrepVacc	2020-2023	Mozambique South Africa, Tanzania, and Uganda (Clade C),	1 668 Adults	DNA/AIDSVAX and DNA/CN54gp140 + MVA/CN54gp140) with PrEP.	—	Pending	(133, 135)

MSM, men who have sex with men; IDUs, intravenous drug users.

## New HIV Vaccine Concepts and Technologies

Vaccines have been used for centuries to prevent and treat various diseases thereby saving millions of lives. Importantly, widespread vaccinations led to the successful eradication of smallpox and significant reduction in other infectious diseases such as polio and measles (136, 137). While modelling research show that a vaccine is essential to conclusively end the HIV epidemic (94), traditional vaccine formulations such as live attenuated and inactivated pathogens and subunit vaccines which offer robust protection against many deadly diseases, are not suitable for HIV vaccines (138). Moreover, cumulative evidence suggests that subunit vaccine designs do not elicit immune responses to levels required for protection, hence the need to utilize novel approaches such as targeted stimulation of broadly neutralizing antibody (bNAb) producing B cell precursors, novel viral vectors and combinatorial vaccine approaches.

### Broadly Neutralizing Antibody (bNAbs) Vaccine Design

The most significant advance in the HIV vaccine field over the past decade has been the identification of bNAbs. Initially, the lack of evidence of antibody mediated suppression of HIV infection *in vivo* led to the belief that the human body was not capable of generating antibodies that can neutralize HIV. The discovery of human sera from HIV infected individuals that could neutralize a broad range of lab-adapted HIV-1 strains led to the rapid isolation and characterization of bNAbs (139–142). From then on, numerous bNAbs have been isolated, some of which can neutralize up to 99% of all known HIV-1 isolates (143). It is now well established that bNAbs responses can be generated during natural infection, but are quite rare and tend to occur much later in chronic infection (144, 145).

Isolation of bNAbs from HIV infected individuals motivated the design of immunogens capable of inducing bNAbs through vaccination. Over the years, bNAbs research has generated deeper insight into the virology and humoral immunity to HIV-1 infection. The wealth of knowledge gained has led to several HIV immunogen design approaches including germ-line targeting immunogens to molecular structural stabilization of envelope trimers such as eOD-GT8 (146, 147), the use of soluble trimers that mimic the native Env spike such as the BG505 SOSI.664 (148), epitope targeted immunogen designs with minimal epitope fragments meant to focus the response on the right region of the Env spike, and minimize off target responses.

It has been shown that all bNAbs typically target the HIV envelope (Env) spike protein (149–151). However, HIV produces numerous non-functional Env proteins which divert antibody immune responses by displaying immunodominant epitopes, resulting in higher titers of non-neutralizing antibody responses (148, 152–154). Immunodominant epitopes tend to be easily accessible whereas most conserved epitopes (vulnerable sites) such as the CD4 binding site (CD4bs) have poor accessibility that limit bNAb recognition (154). One strategy used to increase the immunogenicity of immunodominant epitopes is epitope masking (155). Epitope masking is aimed at directing the immune response to sites of neutralization vulnerability by selectively allowing access to broadly neutralizing epitopes while masking immunodominant (non-neutralizing) regions (156). For instance, addition of glycans to mask immunodominant epitopes has been shown to reduce non-neutralizing antibody access (157, 158).

Another strategy to decrease the immunogenicity of non-neutralizing antibody (non-NAb) epitopes is the occlusion of immunodominant glycan holes. Serological studies conducted in an effort to get insight into the breadth of vaccine-elicited NAbs revealed that immunodominant strain-specific glycan holes on HIV Env contributed to the limited breadth of these monoclonal antibodies (mAbs) (157, 159–162). In addition, it has been shown that the addition of *N*-glycosylation sites to the V3 region or the glycan hole epitope at position 241/289 of the BG505 trimer suppressed the immunogenicity of its non-NAb epitopes while, in some instances, diverting the NAb responses to neoepitopes (163–165). Collectively, these novel strategies provide a platform for the optimization of the epitope targeted immunogen design approaches (97). Furthermore, advances in high throughput recombinant antibody technology has created the possibility of using bNAbs for prevention or treatment of HIV-1 infection as described above (166, 167).

While evidence has shown that cows immunized with BG505 SOSIP trimers rapidly developed broad and potent serum HIV specific NAb responses (168), large-scale bNAb research has not yet achieved the development of a vaccine that induces bNAb responses in other animal models or in humans (169, 170). Deep understanding of host and viral factors necessary and sufficient for the generation of bNAbs is imperative. The extensive Env diversity and the large glycan shield that cover the envelope trimer surface remain a major immunogen design challenge. Moreover, the extremely high somatic hypermutation required for bNAb function makes it very difficult to induce bNAbs by traditional vaccination protocols. To overcome the requirement for high mutation levels, novel approaches that involve priming the initial bNAb precursor B cells followed by sequential immunization aimed at driving the evolutionally intermediates are currently being evaluated in humans (171).

### CMV-Based Vector Vaccines

Clearly, the limited immunogenicity of HIV is still a challenge. Most vaccines induce, weak, narrow, and short-lived immunity. Innovative approaches to overcome this limitation include the use of new viral vectors such as cytomegalovirus (CMV) or Ad26 viral vectors. CMV vectors have emerged as a type of virial vector with unique properties of inducing massive atypical immune responses that are capable of conferring sterilizing protection in animal studies (172). Special attributes of CMV vectored vaccines include the ability to maintain persistent immune stimulation and not being prone to attenuation by pre-existing immune responses. Also, CMV vector-induced HLA-E restricted CD8^+^ T cell responses have the potential to provide vaccine efficacy in all individuals regardless of MHC-class I genotypes (173, 174).

CMV vectors represent a promising strategy in HIV vaccines because of the possibility of genetically reprogramming the vector to induce massive numbers of unusual CD8^+^ T cell responses that can confer sterilizing immunity. The concept of genetic programming of the CMV vector to induce protective immune responses by vaccination was operationalized by Louis Picker and colleagues. In a series of studies, they used non-human primate models to demonstrate that CMV vectors can be genetically calibrated to induce MHC class II restricted as well as non-polymorphic MHC-E restricted CD8^+^ T cell responses. Animals vaccinated with genetically engineered Rhesus CMV (RhCMV/) vectored SIV vaccines elicited very high frequencies of effector memory CD8^+^ T cells that persisted in tissue sites and conferred stringent control of SIV/SHIV in 50% of the vaccinated animals without any apparent antibody responses (175–177).

Although CMV vectors show great promise, safety concerns regarding persistence and potential pathogenicity dampens enthusiasm to use such vaccines in humans. It is also not clear if humans can generate unconventional immune responses reported in animal studies. A study of HIV elite controllers showed that HLA-II restricted CD8^+^ T cell responses exist in humans but are very rare (178), and highly unlikely to be elicited by vaccination. On the other hand, recent studies showed that HLA-E restricted responses are much more common in humans than previously appreciated, which opens up the possibility of inducing such responses by vaccination (179). To improve the safety of CMV vectors, the Picker group has managed to genetically modify CMV vectors to significantly reduce the capacity for the vector to widely disseminate while retaining its ability to superinfect, elicit, and maintain protective CD8^+^ T cell responses (180). These safety improvements render support for testing CMV vectors in humans.

### Combinatorial HIV Vaccine Design

This review has highlighted the inadequacy of standard vaccine approaches to elicit protective immunity against HIV. Therefore, it is imperative that future vaccines adopt multipronged approaches capable of eliciting more than one arm of the immune system. Examples of such approaches for HIV vaccines include the combination of HIV vaccines and non-vaccine approaches such as PrEP, microbicides or other non-vaccine HIV prevention methods. Non-human primate (NHP) studies have provided key proof-of-concept data (181, 182). The first NHP study of combined biomedical preventions (CBP), combined DNA prime recombinant adenovirus boost T cell-based vaccine with a vaginal microbicide gel (with a suboptimal concentration of an HIV-1 nucleocapsid zinc ﬁnger inhibitor) and administered the regime to rhesus macaques (183). CBP delayed simian-human immunodeficiency virus (SHIV) infection in the break through animals and resulted in reduced viral load compared to individual treatments, highlighting strong synergy between the two approaches (183). Another study evaluated the combined effect of a tenofovir-containing vaginal gel co-administered with a protein-based HIV vaccine (containing proteins from clades B and C, adjuvanted with MF59) designed to induce T- and B-cell immunity. The vaccine alone did not offer protection against repeated SHIV_162P3_ challenges, and 1% tenofovir alone showed an efﬁcacy of 46%. But a combination of the vaccine with 1% tenofovir increased protective efficacy to 81% (182). Together, these studies demonstrate the potential of combinatorial approaches to HIV vaccine development and underscore the need for concerted efforts in pursuing such approaches.

## Concluding Remarks

Although considerable progress has been made in understanding the structural and molecular biology of the virus, no HIV vaccine candidate has progressed to licensure, in spite of decades of very expensive research. Nonetheless, there is reason for cautious optimism that at least one of the many vaccine candidates, highlighted in this review, currently in pre-clinical or in efficacy trials will succeed. But, given the history of HIV vaccines, it is not far-fetched to think that success for the current vaccine candidates, if any, is most likely to be modest and will require several iterations to achieve significant protection. The glaring knowledge gap regarding the nature of immunity required for protection warrants basic science research. Given that an effective vaccine will need to stimulate more than one arm of the immune system, we recommend that combinatorial vaccine approaches capable of inducing innate and adaptive immune responses should actively be pursued. Finally, the huge cost of basic research, manufacture and regulatory processes required to take a vaccine candidate from the bench all the way to market, can only be achieved through collaborative efforts from all stake holders. Basic research scientists, vaccine trialists, governments and philanthropists, regulatory bodies, and pharmaceutical companies must continue to pull together to overcome this global challenge.

## Author Contributions

Conceptualization: ZN formulated the idea and provided oversight in planning and organizing the literature search. Writing (original draft preparation) was done by TNg’u and CC with the supervision of ZN. Writing (review and editing): TNg’u, CC, and ZN edited the manuscript. All authors contributed to the article and approved the submitted version.

## Funding

This research was funded by HHMI International Research Scholar award, grant number 55008743 and the NIAID grant number R01A1145305. This work was also funded in part by the Wellcome Strategic Core 201433/Z/16/A Award. Additional funding was provided by the Sub-Saharan African Network for TB/HIV Research Excellence (SANTHE), a DELTAS Africa Initiative [grant # DEL-15-006]. The DELTAS Africa Initiative is an independent funding scheme of the African Academy of Sciences (AAS)’s Alliance for Accelerating Excellence in Science in Africa (AESA) and supported by the New Partnership for Africa’s Development Planning and Coordinating Agency (NEPAD Agency) with funding from the Wellcome Trust [grant # 107752/Z/15/Z] and the UK government. The views expressed in this publication are those of the author(s) and not necessarily those of AAS, NEPAD Agency, Wellcome Trust or the UK government.

## Conflict of Interest

The authors declare that the research was conducted in the absence of any commercial or financial relationships that could be construed as a potential conflict of interest.
